# Comparative analysis of Attention Deficit Hyperactivity Disorder in China and worldwide based on the Global Burden of Disease study

**DOI:** 10.3389/fpsyt.2025.1551672

**Published:** 2025-03-27

**Authors:** Linan Gao, Pengkun Yang, Bo Hu, Yixia Zhang, Zongyuan Liu, Xinruo Wang

**Affiliations:** ^1^ Department of Child Healthcare, Children’s Hospital Affiliated to Zhengzhou University, Zhengzhou, Henan, China; ^2^ Computer Science and Technology Institute, University of Science and Technology of China, Hefei, Anhui, China; ^3^ College of Biological Sciences, China Agricultural University, Beijing, China

**Keywords:** Attention Deficit Hyperactivity Disorder, Global Burden of Disease, disability-adjusted life years, incidence, prevalence, China

## Abstract

**Background:**

Attention Deficit Hyperactivity Disorder (ADHD) is a neurodevelopmental disorder characterized by difficulties in maintaining attention, excessive activity, impulsive behavior, and challenges in organizing and executing tasks. These symptoms can pose challenges in various settings, including home, school, and work, imposing a significant burden not only on affected individuals and their families but also on the global healthcare system.

**Method:**

This study utilized open data from the Global Burden of Disease database covering the years 1990-2021 to analyze the characteristics of the burden of ADHD in China and worldwide, including incidence, prevalence, and adjusted lifespan for disability (DALYs). The Average Annual Percent Change (AAPC) and the corresponding 95% Confidence Interval (95% CI) were calculated using Joinpoint to reflect the trends in ADHD burden. A comprehensive comparative analysis of the differences in the burden of ADHD between China and the world was conducted in multiple dimensions, such as age and gender, using the R software. Statistical significance was indicated by a two-sided P-value of less than 0.05.

**Result:**

From 1990 to 2021, the age-standardized incidence rate (ASIR) of ADHD in China increased from 103.58 per 100,000 to 126.23 per 100,000, while globally, the ASIR decreased from 61.67 per 100,000 to 58.67 per 100,000. The age-standardized prevalence rate (ASPR) in China increased from 1987.98 per 100,000 to 2183.99 per 100,000, contrasting with the global decline from 1228.35 per 100,000 to 1108.89 per 100,000. The age-standardized DALY rate (ASDR) in China also increased from 24.27 per 100,000 to 26.73 per 100,000, however, it decreased from 14.94 per 100,000 to 13.49 per 100,000 globally. The Average Annual Percent Change (AAPC) for ASIR, ASPR, and ASDR in China were 0.64%, 0.28%, and 0.29%, respectively, indicating an upward trend. In contrast, the global AAPC for the burden of ADHD showed a negative trend, with values of -0.16%, -0.34%, and -0.34% for ASIR, ASPR, and ASDR, respectively. The influence of age and gender on the burden of ADHD is distinct, with the highest incidence, prevalence, and DALYs typically observed during childhood. Furthermore, males consistently demonstrate higher rates across these metrics when compared to females.

**Conclusion:**

While there has been a positive development in reducing the burden of ADHD globally, China faces a significant challenge with increasing rates. The focus on childhood and gender differences is crucial for tailored interventions and policies to address ADHD effectively.

## 1 Introduction

Attention Deficit/Hyperactivity Disorder (ADHD) stands as one of the most prevalent neurodevelopmental disorders, with estimates suggesting it impacts 5% – 7.2% of the pediatric population and 2.5% – 6.7% of adults (1). The disorder is marked by symptoms of inattention, hyperactivity, and impulsivity that exceed the normative expectations for an individual’s developmental stage (2). The interplay of genetic predispositions with adverse environmental factors, including prenatal exposures and early life challenges, can contribute to an elevated risk of ADHD manifestation (3, 4). During childhood, individuals with ADHD often exhibit poor interpersonal, parent-child, and sibling relationships, as well as lower academic performance, leading to a lack of self-esteem, low self-evaluation, negative emotions, and other adverse effects (5). In adolescence, ADHD primarily affects learning capabilities, antisocial behavior, the incidence of traffic accidents, and sexual issues (6). In adulthood, individuals with ADHD face numerous challenges in education, occupational functioning, family, and interpersonal relationships (7), and are even at a higher risk for antisocial personality disorders and substance abuse (8). Given the multitude of negative impacts on those affected, as well as the significant economic burden on families and society, ADHD has become a major public health concern. In 2010, researchers for the first time incorporated ADHD into quantitative analyses based on the GBD (9). The most recent update to the GBD database in 2024 has provided us with comprehensive data up to the year 2021, offering a unique opportunity to more precisely delineate the epidemiological characteristics of ADHD. Research indicates that from 1990 to 2019, there has been a varying degree of decline in the age-standardized prevalence and incidence rates of ADHD worldwide (10). ADHD is more common in children, and its incidence appears to increase with socio-economic development. As the largest developing country with the world’s second-largest child population, China may potentially face a unique ADHD burden that could diverge from the global trend of decreasing prevalence. Accordingly, this study conducts a comprehensive analysis and comparison of the burden of ADHD in China and worldwide from 1990 to 2021, based on the most recent GBD data. We employed Joinpoint regression analysis to explore the temporal trends of ADHD, and delved into the variations in burden from the perspectives of age and gender. Our aim is to provide clinicians, patients, and policymakers with more accurate information to address the challenges posed by ADHD, thereby providing a scientific basis for public health policies both globally and within China.

## 2 Materials and methods

### 2.1 Data source and disease definition

The data on ADHD analyzed in this study were sourced from the GBD 2021, accessed through the online query tool of the Global Health Data Exchange (GHDx) platform from the Institute for Health Metrics and Evaluation (IHME). We utilized the GBD tool to extract incidence, prevalence, and DALYs data for ADHD in China and worldwide from 1990 to 2021, employing these as indicators to assess the burden of ADHD. Given that the 2021 GBD data is publicly accessible, the institutional review board granted a waiver for this study, as it was not required to undergo approval. This research adheres to the principles of accurate and transparent health assessment reporting. The GBD 2021 dataset provides the most recent epidemiological estimates for the burden of 371 diseases and injuries across 21 regions and 204 countries and territories from 1990 to 2021 (11). The GBD data is derived from a variety of sources, including national health statistics, censuses, surveys, and research literature. A standardized methodology is employed by GBD to estimate health metrics, ensuring the comparability and consistency of the data. Detailed information regarding the methodology can be found in previous reports (12). Standardized modeling techniques are routinely applied to handle and calculate data specific to diverse population segments, encompassing a spectrum of ages, genders, and geographic locales. Central to our methodology is the utilization of DisMod-MR (13), a pivotal instrument for standardization. As a Bayesian meta-regression tool, DisMod-MR is instrumental in determining the incidence, prevalence, and DALYs associated with ADHD (14). It serves to maintain uniformity across epidemiological metrics, critically appraising the complete array of data at our disposal (15). This rigorous approach is essential for deciphering the evolving landscape of ADHD’s influence on a temporal scale. In the GBD 2021 classification system, which organizes causes into four levels, ADHD is categorized as a Level 3 cause. The hierarchy is as follows: Level 1 for Non-Communicable Diseases, Level 2 for Mental Disorders, and Level 3 specifically for ADHD. The characteristics of ADHD include persistent inattention and/or hyperactive-impulsive behavior manifested across multiple settings, such as at home, in school, or in social situations (16). These symptoms must have been present before the age of 12 and must persist for at least six months. ADHD is defined based on the criteria from the Diagnostic and Statistical Manual of Mental Disorders (DSM, from the third to the fifth edition) or the corresponding category (Hyperkinetic Syndrome) in the International Classification of Diseases (ICD, versions 9 through 10) (10).

### 2.2 Statistical analysis

We specifically extracted ADHD-related metrics from the GBD database for China and globally, covering various age groups and both genders. The indicators we extracted include the age-standardized incidence rate (ASIR), age-standardized prevalence rate (ASPR), and age-standardized disability-adjusted life year rate (ASDR), along with the crude incidence rate (CIR), crude prevalence rate (PR), and crude disability-adjusted life year rate (CDR) for each age group and gender. Using Joinpoint software (National Cancer Institute, Rockville, MD, USA), we incorporated the linear regression model to calculate the Average Annual Percent Change (AAPC) and the corresponding 95% Confidence Interval (95% CI) for assessing the trends in disease burden (17, 18). If the 95% CI of the corresponding AAPC estimate is greater than 0, the age-standardized indicator shows an increasing trend; if it is less than 0, it indicates a decreasing trend; if it includes 0, it suggests a stable trend (19). In addition, to analyze long-term ADHD trends regionally, we used R software program to plot time series of ASIR, ASPR, and ASDR from 1990 to 2021 for China and globally, highlighting epidemiological changes over three decades (20). Furthermore, we analyzed the incidence, prevalence, and DALYs of ADHD across different age groups (categorized in five-year increments) in China and globally, comparing the data from 1990 and 2021. We also compared ADHD-related indicators across different age groups and genders at two time points, and used bar charts to illustrate the differences. The statistical analysis and visualization of the data in this study were conducted using the R statistical software program (version 4.0.3) and the Joinpoint software program (version 4.9.1.0). P value less than 0.05 was considered to be statistically significant.

## 3 Result

### 3.1 Description of the burden of ADHD in China and worldwide

#### 3.1.1 Incidence of ADHD in China and worldwide

The global incidence of ADHD was 3,740,573 cases (95%CI: 2,543,085-5,471,097) in 1990, increasing to 4,111,621 cases (95%CI: 2,775,203-5,954,941) by 2021. Despite the increase in case numbers, the ASIR decreased from 61.67 per 100,000 (95%CI: 41.93-90.19) to 58.67 per 100,000 (95%CI: 39.59-84.99), indicating an average annual percent change (AAPC) of a 0.16% decrease (95%CI: -0.19 to -0.13), showing a downward trend. In contrast, the incidence of ADHD in China showed an upward trend during the same period, with the incidence increasing from 1,134,707 cases (95%CI: 781,431-1,695,760) in 1990 to 1,205,791 cases (95%CI: 822,094-1,743,907) in 2021. The ASIR increased from 103.58 per 100,000 (95%CI: 71.34-154.77) to 126.23 per 100,000 (95%CI: 86.03-182.67), indicating an AAPC of a 0.64% increase (95%CI: 0.60-0.68). This indicates that while the number of global ADHD cases has increased, the standardized rate has shown a downward trend, while the incidence of ADHD in China has been on the rise. (**Table 1**)

**Table 1:** All-age cases and age-standardized incidence, prevalence, and DALYs rates and corresponding AAPC of ADHD in China and globally in 1990 and 2021.

		1990		2021		1990–2021
Location	Measure	All-ages	ASR	All-ages	ASR	AAPE
		n (95% CI)	n (95% CI)	n (95% CI)	n (95% CI)	n (95% CI)
China	Incidence	1134707 (781431-1695760)	103.58 (71.34-154.77)	1205791 (822094-1743907)	126.23 (86.03-182.67)	0.64 (0.60-0.68)
	Prevalence	25506245 (18898184-34816946)	1987.98 (1486.28-2727.83)	24518285 (18634807-33351535)	2183.99 (1638.77-2998.05)	0.28 (0.22-0.33)
	DALYs	311448 (166951-507448)	24.27 (13.07-39.56)	299112 (169780-474761)	26.73 (14.99-42.80)	0.29 (0.23-0.34)
Global	Incidence	3740573 (2543085-5471097)	61.67 (41.93-90.19)	4111621 (2775203-5954941)	58.67 (39.59-84.99)	-0.16 (-0.19- -0.13)
	Prevalence	71433826 (53190050-98854346)	1228.35 (915.031687.76)	84800601 (63395827-117240211)	1108.89 (828.70-1536.23)	-0.34 (-0.36- -0.31)
	DALYs	869478(474087-1424333)	14.94 (8.14-24.49)	1030941 (572105-1670195)	13.49 (7.41-21.89)	-0.34 (-0.36- -0.31)

#### 3.1.2 Prevalence of ADHD in China and worldwide

Between 1990 and 2021, the global prevalence of ADHD showed a declining trend. Despite an increase in the number of cases from 71,433,826 (95% CI: 53,190,050-98,854,346) to 84,800,601 (95% CI: 63,395,827117,240,211), the age-standardized prevalence rate decreased from 1,228.35 per 100,000 people (95% CI: 915.03-1,687.76) to 1,108.89 per 100,000 people (95% CI: 828.70-1,536.23), with an AAPC of -0.34% (95% CI: -0.36 to -0.31). In contrast, the prevalence of ADHD in China showed an upward trend during the same period. The number of cases decreased from 25,506,245 (95% CI: 18,898,184-34,816,946) to 24,518,285 (95% CI: 18,634,807-33,351,535), while the age-standardized prevalence rate increased from 1,987.98 per 100,000 people (95% CI: 1,486.28-2,727.83) to 2,183.99 per 100,000 people (95% CI: 1,638.77-2,998.05), with an AAPC of 0.28% (95% CI: 0.22 to 0.33). This indicates that while the age-standardized prevalence rate of ADHD has decreased globally, it has been on the rise in China. (**Table 1**)

#### 3.1.3 DALYs of ADHD in China and worldwide

From 1990 to 2021, the global ADHD DALYs showed a slight increase in actual numbers from 869,478 (95% CI: 474,087-1,424,333) to 1,030,941 (95% CI: 572,105-1,670,195), yet the age-standardized rate decreased from 14.94 per 100,000 people (95% CI: 8.14-24.49) to 13.49 per 100,000 people (95% CI: 7.41-21.89), with an AAPC of -0.34% (95% CI: -0.36 to -0.31), indicating a decline in the standardized burden of ADHD. In contrast, China’s ADHD DALYs decreased from 311,448 (95% CI: 166,951-507,448) to 299,112 (95% CI: 169,780-474,761), but the age-standardized rate increased from 24.27 per 100,000 people (95% CI: 13.07-39.56) to 26.73 per 100,000 people (95% CI: 14.99-42.80), with an AAPC of 0.29% (95% CI: 0.23 to 0.34), reflecting an increase in the standardized burden of ADHD in China. (**Table 1**)

### 3.2 Joinpoint regression analysis of the burden of ADHD in China and worldwide

**Figure 1:**
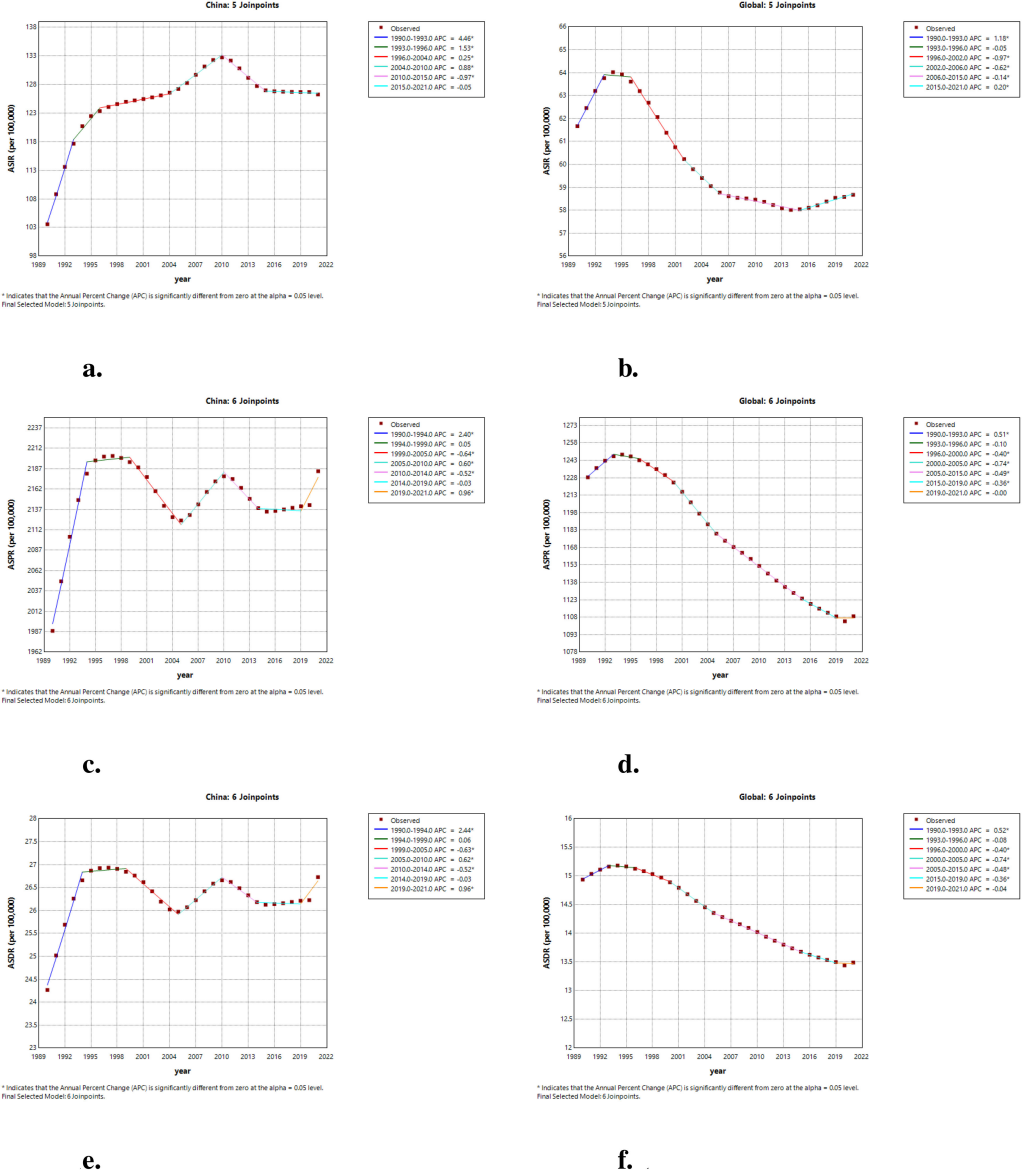
The APC of ASIR, ASPR, and ASDR of ADHD in China and globally from 1990 to 2021. **(a)** The APC of ASIR of ADHD in China; **(b)** The APC of ASIR of ADHD globally; **(c)** The APC of ASPR of ADHD in China; **(d)** The APC of ASPR of ADHD globally; **(e)** The APC of ASDR of ADHD in China; **(f)** The APC of ASDR of ADHD globally (* indicates p-values < 0.05 and significant results).

The annual percent change (APC) of ASIR in China and worldwide from 1990 to 2021 is depicted in **Figures 1a, b**. Incidence in China exhibited an overall upward trend from 1990 to 2004, with an annual growth rate of 4.46% from 1990 to 1993. Although there was a significant decline in incidence between 2010 and 2015, with an APC of -0.97%, the rate of change from 2015 to 2021 was minimal, with an annual percent change close to zero, indicating a stable state. In contrast, the incidence of ADHD worldwide showed a significant increase between 1990 and 1993, followed by a downward trend starting from 1996. Notably, the APC was -0.97% during the period from 1996 to 2002, indicating a substantial decrease. This declining trend persisted until 2006. Comparative analysis reveals that while the global incidence of ADHD was on a clear downward trajectory, incidence in China was still on the rise until after 2010, at which point it began to decline and then stabilized. As shown in **Figures 1c, d**, China saw a rapid increase from 1990 to 1994, a slight decrease until 2005, a rise from 2005 to 2010, and another decline until 2019, followed by a significant increase from 2019 to 2021. In contrast, the global prevalence rose significantly from 1990 to 1993, then slowed and declined steadily from 1996 to 2015, with a minor stabilization from 2015 to 2021. This comparison highlights a distinct trend of prevalence in China compared to the global pattern, with China experiencing an upward trend in recent years. DALYs of ADHD in China rapidly increased from 1990 to 1994, with an annual growth rate of 2.44%, followed by a decline from 1999 to 2005, at an annual rate of -0.63%. Between 2005 and 2010, DALYs rose again, with an annual growth rate of 0.62%, and then decreased from 2010 to 2014, at an annual rate of -0.52%. Notably, China experienced a significant increase in ADHD DALYs from 2019 to 2021, with an annual growth rate of 0.96%. The global ADHD DALYs indicator saw a significant rise from 1990 to 1993, with an annual growth rate of 0.52%, and then began a downward trend starting from 1996, especially between 2000 and 2005, where the annual decrease reached -0.74%. As shown in **Figures 1e, f**, these data highlight the differences in ADHD DALYs between China and the world.

### 3.3 Trends in the burden of ADHD disease in China and worldwide

**Figure 2:**
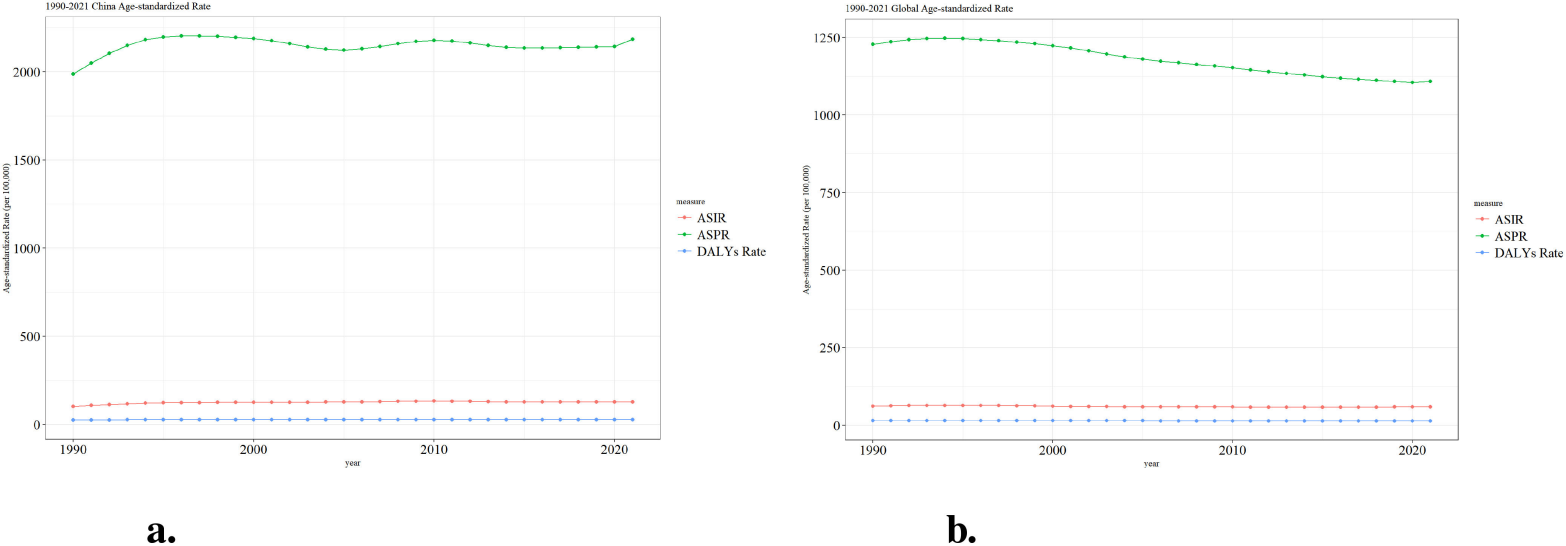
Trend comparison of ASIR, ASPR and ASDR of ADHD in China and worldwide from 1990 to 2021. **(a)** Trend of ASIR, ASPR and ASDR of ADHD in China; **(b)** Trend of ASIR, ASPR and ASDR of ADHD globally.

**Figure 2** presents the trends in age-standardized rates of three health indicators related to ADHD from 1990 to 2021 in China and globally. These indicators include ASIR, ASPR, and DALYs Rate. In China, the ASPR shows an initial increase, followed by a decrease and then stabilization, while the ASIR and DALYs Rate remain relatively low with minimal changes (**Figure 2a**). Globally, the ASPR exhibits a gradual decline, and both the ASIR and DALYs Rate are also maintained at a low level with minor fluctuations (**Figure 2b**). These data reflect the different patterns of ADHD-related health burdens in China and worldwide.

### 3.4 Burden of ADHD in different age groups in China and worldwide in 1990 and 2021

**Figure 3:**
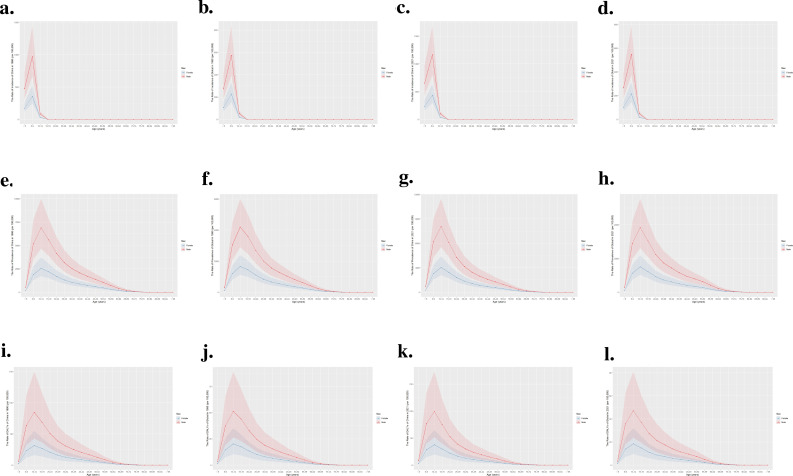
Comparison of the incidence, prevalence, and DALYs rate of ADHD by age group in China and globally for 1990 and 2021. **(a)** Incidence rate in China, 1990; **(b)** Incidence rate globally, 1990; **(c)** Incidence rate in China, 2021; **(d)** Incidence rate globally, 2021; **(e)** Prevalence rate in China, 1990; **(f)** Prevalence rate globally, 1990; **(g)** Prevalence rate in China, 2021; **(h)** Prevalence rate globally, 2021; **(i)** DALYs rate in China, 1990; **(j)** DALYs rate globally, 1990; **(k)** DALYs rate in China, 2021; **(l)** DALYs rate globally, 2021.

**Figure 3** illustrates a comparative analysis of the crude rate of incidence, prevalence, and DALYs associated with ADHD among different age groups in China and globally for the years 1990 and 2021. As shown in **Figures 3a, b**, in 1990, there was a peak in the incidence rate for both males and females during the 5-9 years age group, but the rate for females was significantly lower than that for males. Moreover, the incidence rate in China was higher than the global average. The incidence patterns in 2021, as illustrated in **Figures 3c, d**, mirror those of 1990.

As depicted in **Figures 3e–l**, the data from 1990 and 2021 consistently reveal that males exhibited higher prevalence and DALYs rates than females, especially pronounced in the 5-9 age group. A notable peak in rates for females was observed during the 10-14 age group, followed by a sharp decrease, while males saw the highest rates at 5-9 years of age, which then progressively declined. This pattern was evident both in China and worldwide.

### 3.5 China’s leading role in global ADHD DALYs trends (1990-2021)

**Figure 4:**
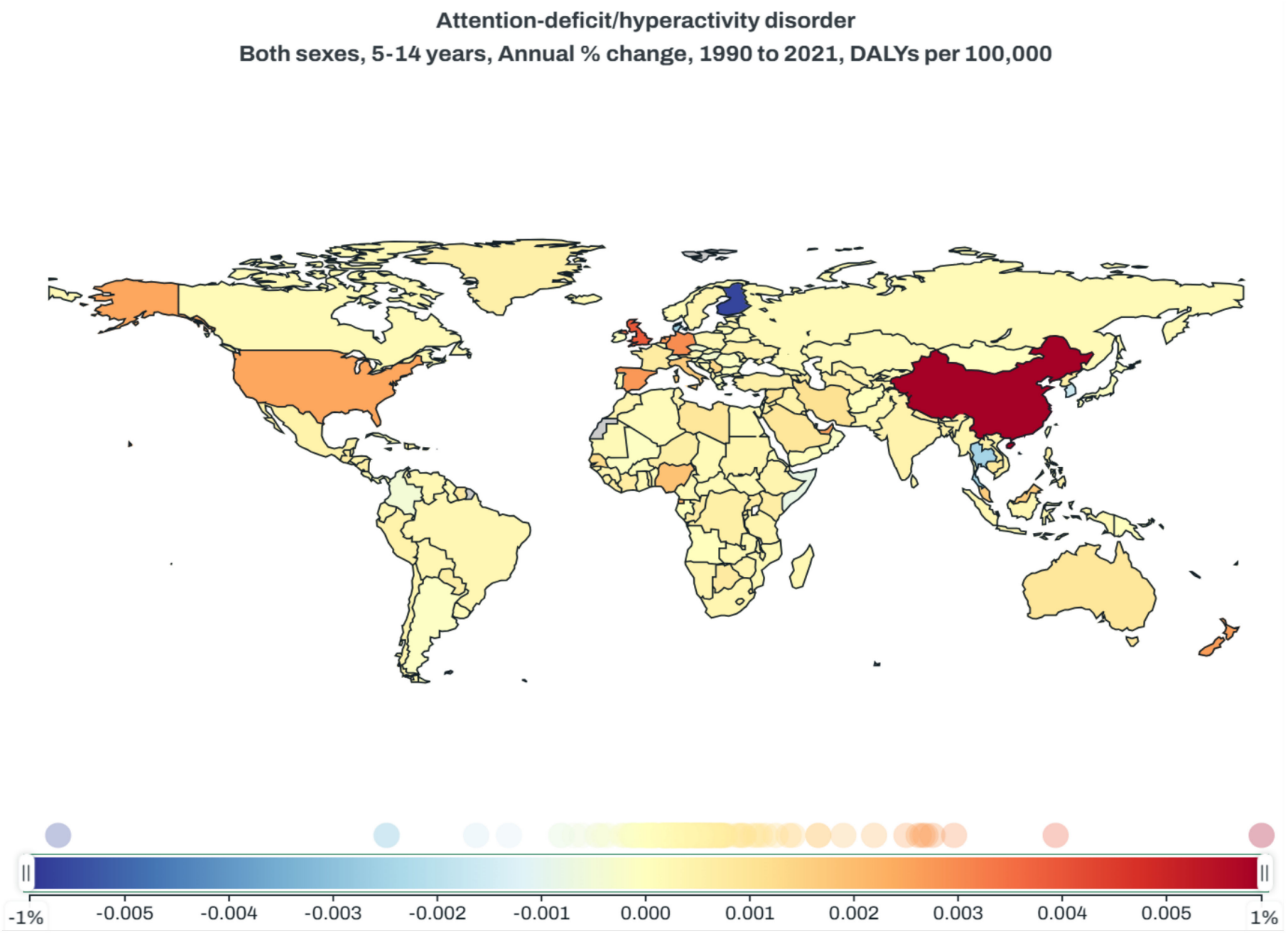
Global map of annual percentage change in ADHD-related DALYs per 100,000 for individuals aged 5-14, 1990-2021.

DALYs is a key metric for assessing the long-term impact of chronic conditions like ADHD on an individual’s overall health and quality of life. It is evident that children aged 5 to 14 years old are the key demographic for ADHD, and for this age group, we have found that from 1990 to 2021, China has been at the forefront globally in terms of the annual percentage change in DALYs associated with ADHD. As is shown in **Figure 4**, the color gradient represents the change in DALYs, with blue indicating a decrease and red indicating an increase. China shows a significant increase, highlighted in dark red.(https://vizhub.healthdata.org/gbd-compare)

### 3.6 Gender disparities in the burden of ADHD in different age groups in China and worldwide

**Figure 5:**
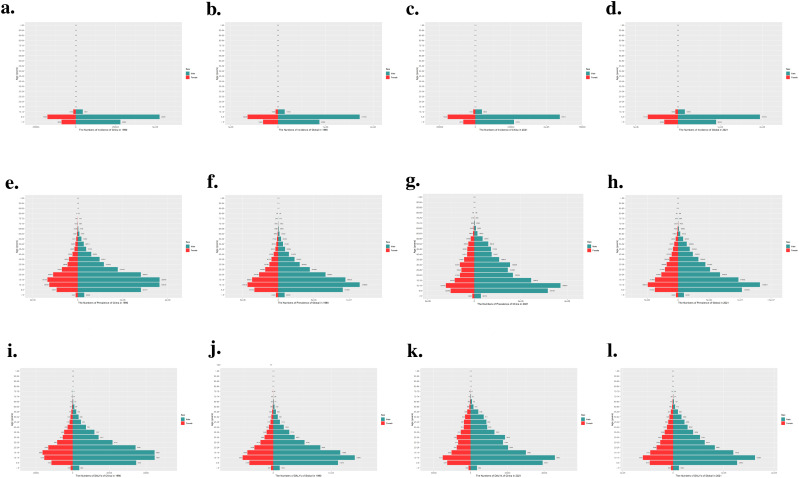
Comparison of the number of incidence, prevalence and DALYs of ADHD in males and females of different age groups in China and globally in 1990 and 2021.

In China and worldwide, in terms of incidence, the gender and age distribution characteristics of ADHD are consistent in both 1990 and 2021. The number of cases in males is higher than in females, and the affected population is only found in those aged less than 5, 5-9, and 10-14 years, with the 5-9 age group being the most prevalent (**Figures 5a–d**). This aligns with the diagnostic criteria for ADHD, which requires symptoms to be present before the age of 12 years old.

As shown in **Figures 5e–h**, in terms of prevalence, both in China and worldwide, males significantly exceed females in both 1990 and 2021. There are significant differences in prevalence across various age groups, with the most affected populations distributed in 5-9 years, 10-14 years, and 15-19 years age groups. The prevalence rate peaks in the 10-14 years age group, after which the number of affected individuals declines with increasing age. In 1990, adolescents aged 15-19 had the second-highest prevalence, but in 2021, the age group with the second-highest prevalence was 5-9 years old. In our study, distribution of DALYs closely follows the prevalence pattern (**Figures 5i–l**): higher prevalence in an age group means more individuals with disabilities, leading to a higher DALYs burden for that group.

### 3.7 Annual ADHD health burden among males and females for China and the global, 1990-2021

**Figure 6:**
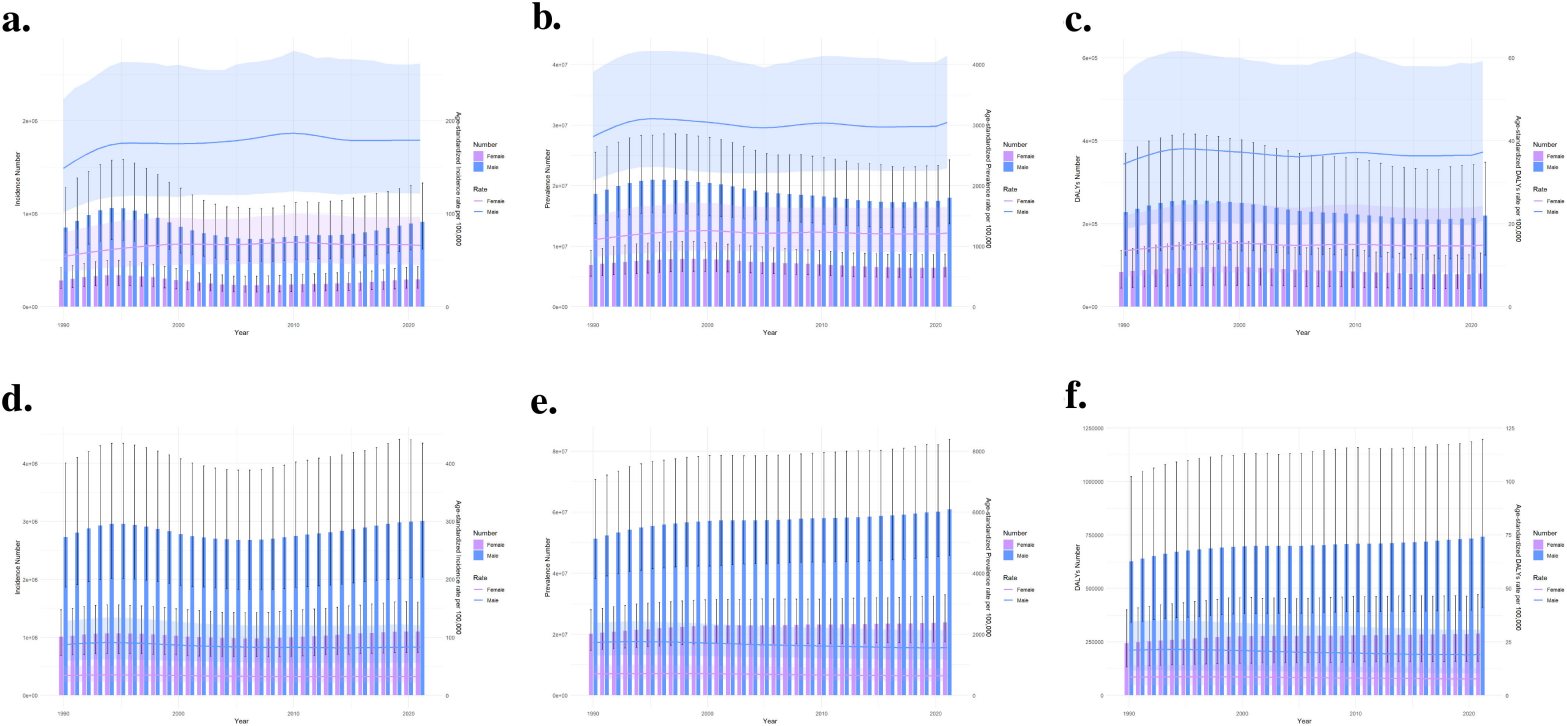
Comparison of full-age cases and age-standardized rates of incidence, prevalence and DALYs among men and women in China and globally from 1990 to 2021.

In China (**Figures 6a–c**), despite annual fluctuations, the rate of incidence, prevalence, and DALYs for ADHD has overall shown a gradual increasing trend, with males consistently exhibiting higher rates than females across all these metrics. In global (**Figures 6d–f**), the burden of ADHD in males is also higher than in females throughout the entire time period. For males, whether it comes to the rate of incidence, prevalence, or DALYs, the figures peaked in the early 1990s, then decreased and stabilized after the year 2000. In contrast, these metrics for females are relatively lower and have shown minimal change, demonstrating a stable trend throughout the period.

## 4 Discussion

The diagnosis of ADHD is a clinical process that involves questionnaire assessments, face-to-face clinical interviews, and neuropsychiatric testing when necessary (1). Geographic location and cultural differences, as well as public health factors such as improved accessibility to medical services, can all influence the incidence and prevalence of ADHD (21). Accurate epidemiological data across time and countries can help test the validity of ADHD diagnoses, approximate the burden associated with the disease, and then make the necessary investments (22). This study, based on the most recently updated GBD database, is the first to conduct an in-depth comparative analysis of the burden and trends of ADHD in China and globally from 1990 to 2021. We comprehensively assessed the incidence, prevalence, and DALYs of ADHD in China and globally from 1990 to 2021. We compared the differences in the burden of ADHD among different age and gender groups in China and globally.

Firstly, combining the results of previous studies and our research, we can conclude that the age-standardized rates of global ADHD incidence, prevalence, and DALYs show a downward trend (23). In contrast, China exhibits an upward trend. It indicates that while there has been positive progress globally in reducing the burden of ADHD, China faces a significant challenge with increasing rates.

The increased burden of ADHD in China may be attributed to several factors. Firstly, rapid socioeconomic development and changes in lifestyle may lead to an increase in ADHD cases (24). Previous studies have indicated that children and adolescents from developed areas have a higher prevalence of ADHD (25). The competitive pressure brought about by economic growth may increase psychological stress in children, potentially inducing or exacerbating ADHD symptoms. The increased consumption of high-sugar and high-fat foods, along with the rise in childhood obesity, may be associated with the increased ADHD symptoms (26). Studies have shown that exposure to environmental pollutants such as particulate matter and harmful gases in the air can lead to negative impacts on the nervous system of children, increasing the risk of ADHD (27). Furthermore, the heightened awareness among parents of children with ADHD can lead to an increased frequency of medical consultations for their children. (28). The genetic factors, as well as the interaction between genetic predisposition and environmental factors, may also play a role in the increasing burden of ADHD (29). The incidence, prevalence, and DALYs of ADHD are related to the age of the patients, being more common in the younger population, with high incidence rates among adolescents (30). Subsequently, as age increases, the incidence rate declines (31). It suggests that we need to focus on the early identification and management of ADHD in children.

In terms of gender composition, males are more commonly diagnosed with ADHD compared to females (32), particularly in pediatric populations, which aligns with previous research (33). This suggests that women with ADHD may be more likely to be overlooked in the diagnostic process, emphasizing the need for gender-specific interventions.

The increase in the burden of ADHD in China has significant implications for public health. It not only places a heavy burden on the affected children and their families but also exerts pressure on the healthcare system. China needs more localized strategies. The success of global initiatives in reducing the burden of ADHD may provide valuable insights for China to adapt and implement similar strategies tailored to its unique socio-cultural and economic context.

It is important to acknowledge the limitations of this study. Firstly, the accuracy of GBD research results largely depends on the quality and completeness of the data relied upon (34), and there is often a lack of adequate reporting and diagnosis for ADHD. Secondly, the existing data do not provide sufficient detail to analyze the severity and treatment response of ADHD, as this information is not recorded in the GHDx database. Lastly, as this analysis uses observational data, unidentified confounding variables may restrict our ability to establish causality in the observed trends (35). Future research should focus on understanding the specific factors contributing to the increasing burden of ADHD in China and evaluate the effectiveness of interventions aimed at reducing this burden.

In conclusion, the comparative analysis of ADHD burden between China and the global reveals significant differences in trends and highlights the need for targeted interventions. Understanding these disparities is crucial for developing effective public health policies and strategies to address the challenges posed by ADHD.

## Data Availability

Publicly available datasets were analyzed in this study. This data can be found here: http://ghdx.healthdata.org/gbd-results-tool.
